# Capillary refill time response to a fluid challenge or a vasopressor test: an observational, proof-of-concept study

**DOI:** 10.1186/s13613-024-01275-5

**Published:** 2024-04-01

**Authors:** Glenn Hernández, Emilio Daniel Valenzuela, Eduardo Kattan, Ricardo Castro, Camila Guzmán, Alicia Elzo Kraemer, Nicolás Sarzosa, Leyla Alegría, Roberto Contreras, Vanessa Oviedo, Sebastián Bravo, Dagoberto Soto, Claudia Sáez, Hafid Ait-Oufella, Gustavo Ospina-Tascón, Jan Bakker

**Affiliations:** 1 Departamento de Medicina Intensiva, Facultad de Medicina, Pontificia Universidad Católica de Chile, Avenida Diagonal Paraguay 362, Santiago, Chile; 2 Departamento de Hematología Oncología, Facultad de Medicina, Pontificia Universidad Católica de Chile, Santiago, Chile; 3 Medical Intensive Care Unit, Hôpital Saint-Antoine, Assistance Publique-Hôpitaux de Paris, Sorbonne Université, Paris, France; 4 Cardiovascular Research Center, INSERM U970, Université de Paris, Paris, France; 5 Department of Intensive Care Medicine, Fundación Valle del Lili, Cali, Colombia; 6 Translational Research Laboratory in Critical Care Medicine (TransLab-CCM), Universidad Icesi, Cali, Colombia; 7 Department of Intensive Care Adults, Erasmus MC University Medical Center, Rotterdam, Netherlands

**Keywords:** Capillary refill time, Septic shock, Fluid challenge, Vasopressor test, Perfusion.

## Abstract

**Background:**

Several studies have validated capillary refill time (CRT) as a marker of tissue hypoperfusion, and recent guidelines recommend CRT monitoring during septic shock resuscitation. Therefore, it is relevant to further explore its kinetics of response to short-term hemodynamic interventions with fluids or vasopressors. A couple of previous studies explored the impact of a fluid bolus on CRT, but little is known about the impact of norepinephrine on CRT when aiming at a higher mean arterial pressure (MAP) target in septic shock. We designed this observational study to further evaluate the effect of a fluid challenge (FC) and a vasopressor test (VPT) on CRT in septic shock patients with abnormal CRT after initial resuscitation. Our purpose was to determine the effects of a FC in fluid-responsive patients, and of a VPT aimed at a higher MAP target in chronically hypertensive fluid-unresponsive patients on the direction and magnitude of CRT response.

**Methods:**

Thirty-four septic shock patients were included. Fluid responsiveness was assessed at baseline, and a FC (500 ml/30 mins) was administered in 9 fluid-responsive patients. A VPT was performed in 25 patients by increasing norepinephrine dose to reach a MAP to 80–85 mmHg for 30 min. Patients shared a multimodal perfusion and hemodynamic monitoring protocol with assessments at at least two time-points (baseline, and at the end of interventions).

**Results:**

CRT decreased significantly with both tests (from 5 [3.5–7.6] to 4 [2.4–5.1] sec, *p* = 0.008 after the FC; and from 4.0 [3.3–5.6] to 3 [2.6 -5] sec, *p* = 0.03 after the VPT. A CRT-response was observed in 7/9 patients after the FC, and in 14/25 pts after the VPT, but CRT deteriorated in 4 patients on this latter group, all of them receiving a concomitant low-dose vasopressin.

**Conclusions:**

Our findings support that fluid boluses may improve CRT or produce neutral effects in fluid-responsive septic shock patients with persistent hypoperfusion. Conversely, raising NE doses to target a higher MAP in previously hypertensive patients elicits a more heterogeneous response, improving CRT in the majority, but deteriorating skin perfusion in some patients, a fact that deserves further research.

**Supplementary Information:**

The online version contains supplementary material available at 10.1186/s13613-024-01275-5.

## Introduction

Several clinical-physiological studies have validated capillary refill time (CRT) as a marker of tissue hypoperfusion particularly at the skin level, an extensive territory where microcirculatory flow is primarily affected by the compensatory neurohumoral response to shock [1–12]. CRT is directly related to skin blood flow [5] and microvascular reactivity [5, 9–11], and eventually to intraabdominal organ perfusion [8]. Therefore, recent guidelines have recommended CRT monitoring as a perfusion variable during septic shock resuscitation [12].

From an epidemiological perspective, a robust number of observational studies have demonstrated that achieving a normal peripheral perfusion after initial septic shock resuscitation is associated with a mortality risk of less than 20% as compared to more than 40% in patients with persistently abnormal CRT [1, 3, 5, 13, 14]. In addition, a recent randomized controlled trial (RCT) found that targeting CRT during early septic shock resuscitation resulted in less organ dysfunction and a trend to lower mortality when compared with lactate-guided resuscitation [15, 16]. A post-hoc analysis of the same trial found that patients that had normalized CRT at two hours (CRT-responders) exhibited a significant lower mortality than those with persistent abnormal CRT (non-responders) [2]. Thus, CRT normalization may signal a successful resuscitation in shock states, although the precise pathophysiological link is not yet fully understood [13].

The previous considerations position CRT as a potential resuscitation target, and therefore, it appears as relevant to further explore its kinetics of response to common hemodynamic interventions such as fluids or vasopressors. Two recent small physiological studies found that CRT improves in minutes after a fluid bolus in most fluid-responsive patients, although not in all, a fact that may be attributed to the background status of macro-microcirculatory coupling, among other factors [6, 13, 17]. These studies included some patients with normal CRT at baseline and variable response criteria.

Little is known, however, on the impact of the impact of different doses of vasopressors, particularly norepinephrine (NE) on CRT. This appears as particularly relevant in the context of the controversy on the best mean arterial pressure (MAP) target in septic shock, since targeting a higher MAP necessarily implies raising NE doses, an action that has demonstrated conflicting effects on sublingual microcirculation depending on the basal status of the microcirculation [18, 19]. The ANDROMEDA-SHOCK Trial introduced the vasopressor test (VPT), a transient increase in NE doses to reach a higher MAP target in previously hypertensive septic shock patients with persistent hypoperfusion [15]. The VPT test was used in 28% of the patients in the CRT arm with a success rate of 40% one hour later. A more recent clinical physiological study showed that doubling the dose of NE produced no consistent effect on CRT [17].

We designed this observational study to further evaluate the effect of a fluid challenge (FC) and a VPT on CRT in septic shock patients with persistent abnormal CRT after initial resuscitation. Our purpose was to confirm the effects of a fluid challenge on CRT in fluid-responsive patients. Additionally, we aimed at exploring the effect of a VPT with NE on the direction and magnitude of CRT response to the test in previously hypertensive fluid-unresponsive patients.

For this purpose, we selected septic shock patients from three clinical studies that assessed, as part of their objectives and design, CRT response to a standardized FC or a VPT and shared a set of pre- and post-intervention measurements of clinical, hemodynamic, and perfusion-related variables.

## Methods

### Background studies

**Fig. 1:**
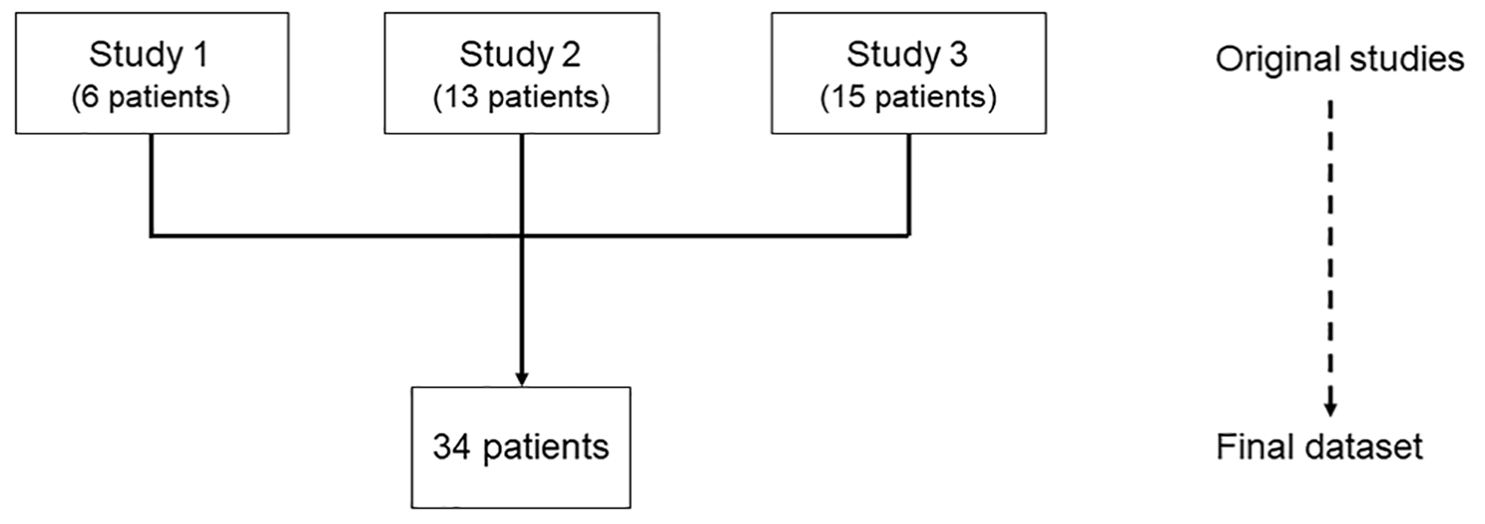
Study Flow

Patients were selected as a convenience sample from the three background studies (two RCT´s and one observational) conducted at an academic medical center from October 2020 to June 2023 (Fig. 1 and ESM Table 1).

The three studies were approved by the local Institutional Review Board (Comité de Ética Asistencial, Facultad de Medicina, Pontificia Universidad Católica de Chile; Numbers 190,527,001, 200,318,004 and 221,107,002, respectively), and were supported by ANID Chile (FONDECYT grants 1,200,246 and 11,201,220). All patients or their next of kin signed an informed consent to be included in both RCTs (NCT 04693923, NCT 06125184) but this requirement was waived in the third one because of its observational, non-invasive design.

### Population

Septic shock [20] patients under mechanical ventilation, with a stable MAP ≥ 65 mmHg and an abnormal CRT after initial resuscitation were eligible for the present study.

Patients were excluded if they had one of the following conditions: active hemorrhage, severe acute respiratory distress syndrome, do-not-resuscitate status, pregnancy, or more than 12 h of septic shock evolution. In addition, patients were excluded if assessments of CRT, cardiac output (CO), or fluid-responsiveness (FR) were not feasible.

All patients were receiving NE as the primary vasopressor drug, but a concomitant low-dose vasopressin (0.02 units/min) was acceptable since it is a common practice in our unit, provided that it was not modified during the tests.

### Common study procedures

Baseline clinical characteristics, severity, and hemodynamic and perfusion status were recorded.

After registering the baseline parameters, FR was assessed, and a standard FC or a VPT was performed depending on FR status. FR was determined using different techniques according to the clinical context [21, 22].

- Fluid challenge: a FC was used only in fluid-responsive patients with a standard 500 mL of crystalloid bolus administered in 30 min. NE dose was not modified during the test either unless for safety reasons such as a fall in MAP below 60 mmHg.
- Vasopressor test: Only fluid unresponsive patients with chronic hypertension were subjected to a VPT. MAP was transiently increased to 80–85 mmHg by raising NE doses. The post- MAP intervention assessments were performed 30 min after reaching the MAP goal of 80–85 mmHg.

Patients shared a multimodal perfusion and hemodynamic monitoring protocol with assessments at at least two time-points (baseline, and at the end of the standard FC or 30 min post-MAP intervention). Multimodal monitoring included hemodynamic and perfusion variables, CRT, vasoactive and fluid administration. CO was measured with non-calibrated devices such as Argos Monitor ® (Retia Medical, USA), or Flo Trac Monitor® (Edwards Life Sciences, USA).

### Capillary refill time

CRT was determined with a standardized technique described elsewhere [21]. Briefly, CRT was measured by applying firm pressure to the ventral surface of the right index finger distal phalanx with a glass microscope slide. The pressure was increased until the skin was blank and maintained for 10 s. The time for return of the normal skin color was registered with a chronometer, and a CRT > 3 s was defined as abnormal.

We included only patients with an abnormal CRT at baseline and defined CRT response as a decrease of ≥ 1 s after the intervention since this appears to be a clinically measurable and potentially relevant change.

### Statistical analysis

The Kolmogorov-Smirnov test was used to assess distribution normality for each variable. Descriptive statistics are presented as mean +- standard deviation, median [interquartile range] or percentage. Students’ t-test, Mann–Whitney U Test, paired t-test, Wilcoxon signed-rank test, chi-square or Fisher’s exact test were used when appropriate. Two-tailed *p* value < 0.05 was considered significant. Data were analyzed with Minitab v17 (Minitab Inc, State College, PA) and Graphpad Prism v10.0 (Graphpad Softwares, La Joya, CA) softwares.

## Results

Thirty-four septic shock patients were included in this study (Fig. 1). Baseline characteristics are shown in Table 1.

**Table 1:** Baseline characteristics of the study population

	Value
**Age** (years**)**	71 [65–77]
**Female**	53%
**BMI** (kg/m^2^)	27.8 ± 7.8
**APACHE**	18.3 ± 8.3
**SOFA**	9.2 ± 2.5
**CRP** (mg/dL)	24.1 ± 15.2
**Sepsis Source**	
*Abdominal*	55% (19)
*Respiratory*	24% (8)
*Urinary*	9% (3)
*Soft tissue*	6% (2)
*Other*	6% (2)
**Previous fluid resuscitation** (mL)	1610 [670–2868]
**NE dose** (mcg/kg/min)	0.19 ± 0.15
**Vasopressin use** (%)	17%
**MAP** (mmHg)	67 [64–70]
**HR** (bpm)	92 ± 18
**CRT** (s)	4 [3.1-6]
**Lactate** (mmol/L)	3 [2.3–4.3]
**CVP** (mmHg)	8.6 ± 4.8
**Cardiac output** (L/min)	4.9 ± 1.7
**Central venous O**_**2**_**saturation** (%)	77 [69–84]
**Venous-arterial pCO**_**2**_ **gradient**	4.4 ± 2.5

BMI: Body Mass Index; APACHE: Acute Physiology and Chronic Health Evaluation; SOFA: Sequential Organ Failure Assessment; CRP: c-reactive protein; NE: norepinephrine; MAP: mean arterial pressure; HR: heart rate; CRT: capillary refill time; CVP: central venous pressure. Data is presented as mean ± SD or median [interquartile range]

Diverse tests were used to assess FR including pulse pressure variation with a tidal volume challenge 79%, and passive leg raising with velocity-time integral (VTI) assessment 12%, while in 9% other tests were used. One patient in the FC subgroup and five in the VPT subgroup were receiving a basal infusion of vasopressin at a fixed dose of 0.02 units/min.

A standard FC was administered in nine fluid-responsive patients, and 25 fluid-unresponsive patients were subjected to a VPT.

Pre-and post-intervention data are shown in Table 2. On average, CRT decreased significantly with both tests (from 5 [3.5–7.6] to 4 [2.4–5.1] sec, *p* = 0.008 after the FC; and from 4.0 [3.3–5.6] to 3 [2.6 -5] sec, *p* = 0.03 after the VPT. CO increased significantly after the FC from 5.7 ± 1.7 to 6.3 ± 2.0 (L/min), *p* = 0.03. MAP increased with the VPT from 67 [64–70] to 84 [82–87] mmHg, *p* = 0.0001.

**Table 2:** Macrohemodynamic and perfusion variables before and after the hemodynamic interventions

	Pre-intervention	Post-intervention	*p*-value
**Fluid challenge**			
**CRT** (s)	5 [3.5–7.6]	4 [2.4–5.1]	0.008
**MAP** (mmHg)	66 [62–79]	72 [62–75]	0.96
**SAP** (mmHg)	101 [88–112]	102 [97–108]	0.99
**DAP** (mmHg)	56 [48–64]	52 [45–60]	0.17
**Pulse Pressure** (mmHg)	50 [39–56]	50 [37–55]	0.66
**HR** (bpm)	103 ± 24	104 ± 25	0.72
**NE Dose** (mcg/kg/min)	0.31 ± 0.17	0.35 ± 0.24	0.26
**CVP** (mmHg)	7.6 ± 4.4	5.9 ± 4.5	0.04
**CO** (L/min)	5.7 ± 1.7	6.3 ± 2.0	0.03
**SV** (ml)	56 ± 10	59 ± 7	0.2
***Vasopressor test***			
**CRT** (s)	4.0 [3.3–5.6]	3 [2.6 -5]	0.03
**MAP** (mmHg)	67 [64–70]	84 [82–87]	0.0001
**SAP** (mmHg)	106 [96–118]	132 [120–142]	0.0001
**DAP** (mmHg)	47 [45–54]	57 [53–60]	0.0001
**Pulse Pressure** (mmHg)	55 [42–67]	69 [62–87]	0.002
**HR** (bpm)	88 ± 15	86 ± 18	0.17
**NE Dose** (mcg/kg/min)	0.18 ± 0.13	0.28 ± 0.19	0.001
**CVP** (mmHg)	9.2 ± 5	8.8 ± 4.7	0.9
**CO** (L/min)	4.7 ± 1.4	5.0 ± 1.7	0.15
**SV** (ml)	55 ± 17	59 ± 18	0.03

CRT: capillary refill time; MAP: mean arterial pressure; SAP: systolic arterial pressure; DAP: diastolic arterial pressure; HR: heart rate; NE: norepinephrine; CVP: central venous pressure; CO: cardiac output; SV: stroke volume. Data is presented as mean ± SD or median [interquartile range]. Paired t-test or Wilcoxon sum-rank test used accordingly

**Fig. 2:**
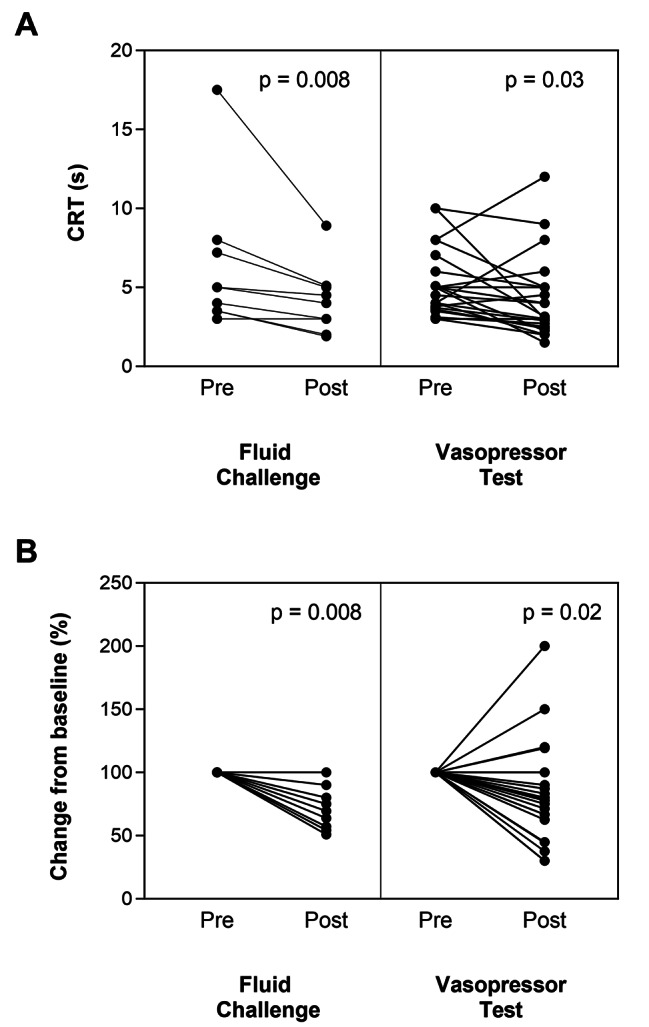
Pre- and post-intervention measurements of CRT after either a fluid challenge or and a vasopressor test. Panel **A** shows absolute change while panel **B** shows relative changes of CRT.

Individual changes in absolute values of CRT after interventions are depicted in Fig. 2 Panel A. Panel B shows the relative change from baseline for both tests. A standard FC in fluid-responsive patients improved CRT in 7/9 patients, while no patient worsened skin perfusion. NE dose had to be slightly increased during the FC in two patients after registering a MAP < 60 mmHg.

Conversely, a VPT improved CRT in 14/25 patients, but CRT deteriorated in four patients. These 4 patients were on concomitant vasopressin infusion. Of these, three patients worsened CRT by more than one second (8 to 12 s; 4 to 8 s; 5 to 6 s) and one from 3.8 to 4.5 s.

A comparison of some clinical characteristics, hemodynamic and perfusion variables at baseline between CRT responders and non-responders in the VPT subgroup is shown in Table 3. CRT responders exhibited lower CO and NE requirements at baseline.

**Table 3:** Comparison of different clinical, hemodynamic and perfusion variables at baseline in CRT-responders versus non-responders to the vasopressor test

	Responders	Non-responders	*p*-value
**Number of patients**	14	11	
**Age** (years)	76 [66–81]	68 [66–78]	0.3
**APACHE**	20 ± 9.3	19.6 ± 5.7	0.9
**SOFA**	9.3 ± 2.4	9.4 ± 2.6	0.34
**CRP** (mg/dL)	31.1 ± 14.5	20.4 ± 16.3	0.004
**CO** (L/min)	3.9 ± 1.2	5.5 ± 1.3	0.047
**NE dose** (mcg/kg/min)	0.13 ± 0.11	0.24 ± 0.13	0.12
**CVP** (mmHg)	7.4 ± 4.4	11 ± 5.1	0.7
**HR** (bpm)	86 ± 15	89 ± 16	0.056
**CRT** (s)	5.0 [3.8–6.5]	3.7 [3–5]	0.49
**Lactate** (mmol/L)	2.5 [2-3.3]	3.8 [1.6–8.8]	0.1
**Central venous O2 saturation** (%)	69 [65–84]	82 [74–85]	0.7
**Venous-arterial pCO2 gradient** (mmHg)	3 [1–4]	3 [3–6]	0.8

APACHE: Acute Physiology and Chronic Health Evaluation; SOFA: Sequential Organ Failure Assessment; APACHE: Acute Physiology and Chronic Health Evaluation; CRP: c-reactive protein; CO: cardiac output; NE: norepinephrine; CVP: central venous pressure; HR: heart rate; CRT: capillary refill time. Data is presented as mean +- SD or median [interquartile range]. Students’ t-test or Mann-Whitney U-test used accordingly

## Discussion

Our main findings can be summarized as follows: In a cohort of septic shock patients with abnormal CRT after initial resuscitation, CRT decreased significantly in response to two standardized hemodynamic tests, a fluid challenge and a vasopressor test. Most patients showed a positive CRT response after the FC test, with no cases of CRT worsening in this subgroup. Conversely, the VPT elicited a more heterogeneous response, improving CRT slightly over half of patients, while a subset experienced significant worsening. These latter were under a concomitant low-dose vasopressin infusion. Our findings add interesting data to further characterize the effect of common hemodynamic interventions on CRT and tend to confirm its rapid response in a substantial number of patients, thus further supporting its potential role as a bedside monitor during septic shock resuscitation. The finding of CRT deterioration after increasing NE doses in the presence of a concomitant low-dose vasopressin requires further exploration.

Several recent studies support the use CRT to assess the short-term impact of acute hemodynamic interventions [6, 17, 23]. Jacquet-Lagreze et al. found that PLR-induced changes in CRT accurately predicted CRT-response to a fluid bolus in 34 patients with acute circulatory dysfunction, and overall, only 44% of patients were CRT-responders [23]. On the other hand, Raia et al. evaluated the kinetics of CRT response after a fluid challenge in 40 septic patients [6]. 79% of patients were CRT-responders and among them CRT rapidly improved with a significant decrease at 6–8 min that was maintained at 30 min. More recently, Fage et al. evaluated the acute effect of a fluid bolus and a NE dose increase on CRT in 69 septic shock patients [17]. Significant changes in CRT, were found only in patients with abnormal CRT at baseline and with increases > of 15% in CO or > 15% in MAP after the hemodynamic interventions. However, even among this subgroup, CRT response was variable, while decreasing in some but remaining stable in others [17]. The criteria used to define CRT response in these three studies were diverse ranging from a decrease of > 23% to just > 0.2 s. Conversely in our model, (i) we included only septic shock patients with an abnormal CRT at baseline; (ii) we defined a decrease of ≥ 1 s as a significant response criterion; and (iii) as a difference with Fage’s study, we considered for VPT analysis only previous fluid-unresponsive chronic hypertensive patients in whom a standardized test was applied.

Concerning CRT-response to the fluid challenge in our population, 77% of the patients exhibited a significant CRT decrease after a standardized fluid bolus. As expected, this response was associated with a significant increase in CO. On the other hand, the same as in previous studies [6, 17] some patients were non-responders, but no one deteriorated CRT after the FC.

Our findings concerning CRT-response to a vasopressor test are particularly interesting considering the ongoing controversy on the best MAP target for chronically hypertensive patients with septic shock [24–27]. Indeed, two large RCTs addressing this issue found conflicting effects on major outcomes when targeting different levels of MAP and the issue is far from settled [26, 27]. Current guidelines recommend starting with a 60–65 mmHg MAP target and then individualize according to perfusion response although with no clear proposal on how to implement this recommendation [12].

From a physiological point of view, the extent to which increased MAP contributes to improvement of microcirculatory perfusion is variable, depending on the balance between the increase in systemic organ perfusion pressure versus a potential impairment at the microcirculatory driving pressure level [28]. In fact, Thooft et al. [18] and Dubin et al. [19] found that changes in sublingual microvascular flow after increasing MAP with NE in septic shock patients were highly heterogeneous since patients with a lower microvascular flow at baseline tended to improve flow at higher MAP levels, while the inverse occurred in those with a normal one.

Since assessment of sublingual microcirculation is only a research tool, CRT appears as a physiologically sound surrogate [13]. The ANDROMEDA-SHOCK trial introduced the concept of VPT, meaning a transient increase in MAP levels to 80–85 mmHg in chronically hypertensive fluid-unresponsive septic shock patients with persistent hypoperfusion, and evaluating response after a short period of time [15]. 28% of patients in the CRT arm required the VPT with a positive CRT-response in 44%.

CRT response to a higher MAP level was not as successful as with a fluid challenge but a proof of benefit in CRT-responders may aid clinicians in deciding to maintain this higher MAP target. As or more importantly, the real-time detection of patients in whom increasing vasopressor doses impairs tissue perfusion is a very important clue for deciding on a lower MAP goal. Intriguingly, this occurred in patients with a concomitant low-dose vasopressin, a fact that in our opinion, raises concerns and deserves further exploration. In other words, if confirmed by further studies, this practical bedside VPT may contribute to shed light into the best MAP target controversy [24–27] and provide objective data to personalize MAP goals in septic shock patients.

Our study has several limitations. First, it is observational, but only patients subjected to a standardized FC or a VPT and that shared a set of pre- and post-intervention measurements of clinical, hemodynamic, and perfusion-related variables, were included. Second, only one test per patient was performed. Conducting both tests on the same patients could have provided more comprehensive insights into the interplay between different hemodynamic interventions and CRT response. Third, it does not include other independent microcirculatory assessments as planned in the original studies mainly because of pandemic-related shortage of specific technical devices. Fourth, the inclusion of patients with concomitant vasopressin infusion is debatable and should be probably avoided in future studies. Fifth, the administration of a fluid challenge in 30 min is probably too long under current knowledge and may confound interpretation of results. Sixth, the use of non-calibrated CO monitors precluded us to explore in more depth the relationship between CO changes and CRT response. Finally, the observational nature and limited sample size of our study call for a cautious interpretation and highlight the necessity for larger, more comprehensive studies to confirm and extend our findings.

## Conclusions

Our findings support that fluid boluses may improve CRT in fluid-responsive septic shock patients with persistent hypoperfusion, while producing neutral effects in a few. Conversely, raising NE doses to target a higher MAP in previously hypertensive patients elicits a more heterogeneous response, improving CRT in the majority, but deteriorating skin perfusion in some patients with concomitant vasopressin infusion, a fact that deserves further research. Assessing CRT response to acute hemodynamic interventions, such as described in this study, may potentially aid in the effort to personalize septic shock resuscitation.

### Electronic supplementary material

Below is the link to the electronic supplementary material.

Supplementary Material 1

## Data Availability

The datasets are available from the corresponding author on reasonable request.
