# Supplementary material for: Capillary refill time response to a fluid challenge or a vasopressor test: an observational, proof-of-concept study
Source: Ann Intensive Care. 2024 Apr 1;14:49. doi: 10.1186/s13613-024-01275-5 (PMC10984906; doi:10.1186/s13613-024-01275-5)
Supplement: Supplementary file 1 — Supplementary Material 1 [file 13613_2024_1275_MOESM1_ESM.docx]

**ESM Table 1. Background research projects for the present study**

**Study one:** Capillary Refill Time Response to a Rapid Fluid Challenge in Septic Shock Patients (NCT 04693923, ANID grant FONDECYT 1200246): Six patients included.

**Study two:** Effect of Vasopressin on Kidney and Cardiac Function in Septic Shock

(NCT 06125184, ANID grant FONDECYT11201220): Thirteen patients included.

**Study three:** Prospective observational study on the impact of different hemodynamic interventions on CRT in critically ill patients: 15 patients included.
